# Editorial: Adult ADHD and other psychiatric disorders

**DOI:** 10.3389/fpsyt.2022.1058117

**Published:** 2022-11-01

**Authors:** Francesco Oliva, Giulia di Girolamo, Luana Salerno, Stefano Pallanti, Laura Wilson, Alexandra Philipsen

**Affiliations:** ^1^Department of Clinical and Biological Sciences, University of Turin, Turin, Italy; ^2^Department of Neuroscience, University of Turin, Turin, Italy; ^3^Institute of Neuroscience, Florence, Italy; ^4^Department of Psychiatry and Psychotherapy, University Hospital Bonn, Bonn, Germany

**Keywords:** attention deficit and hyperactivity disorder, comorbidiity, brain structural abnormalities, subjective perception of disturbance, sexuality

Attention deficit and hyperactivity disorder (ADHD) is one of the most common neurodevelopmental disorders. Its onset is typically in childhood, but it persists very frequently into adulthood. Although the deleterious impact of its core and accessory symptoms on all the individual's functioning areas is well-known, ADHD can remain underdiagnosed in adults also because of the high rate of co-occurrence with other psychiatric disorders (1). Accessory symptoms such as late-onset sleep, low self-esteem, and emotional dysregulation are indeed often misinterpreted as clinical manifestations of mood, anxiety, and personality disorders (2).

The present research topic aimed to deepen ADHD issues in adults considering the influence of other psychiatric disorders that commonly lead to complex psychopathology and clinical presentation.

As editors of this Research Topic, it was our pleasure to review several papers. Among these, the following four articles responded to the topic aim, encompassing different points of view, ranging from neuroimaging to patients' subjective perception of ADHD core and accessory symptoms.

As regards symptoms overlapping, in their thorough fMRI study, Gerhardt et al. specifically compared ADHD impulsivity with that of alcohol use disorder. The authors examined the subprocesses that constitute the response inhibition (i.e., interference inhibition, action withholding, and action cancellation) by scanning cerebral regions while patients and controls were performing a hybrid response inhibition task. Results confirm that impulsivity is a common phenotype in these two diagnoses, and also shed light on different functional impairments underpinning this characteristic in those two clinical conditions.

On the other hand, Van Rooij et al. focused on morphological brain markers that may distinguish adult patients between those who present ADHD alone and those who are also affected by reward-related comorbidities (i.e., major depressive disorder, substance use disorder, and obesity). They conducted a comprehensive neuroimaging investigation, finding no significant difference in cortical thickness in the medial, dorsal, and frontal brain areas. These findings suggest that brain morphometry cannot help disentangling the complex relationship between ADHD and these comorbidities at a biological level.

The study by Hertz et al. is one of the few considering a variety of sexual behaviors among adult patients with ADHD in comparison with non-ADHD controls. Furthermore, the authors explored the relationship between ADHD symptoms and sexual behaviors (including sexual risk-taking) as well as sexual dysfunctions. Attention deficit and hyperactivity disorder patients of both sexes reported higher rates of sub-clinical hypersexual behaviors than controls, but unexpectedly no differences emerged concerning risky sexual behaviors and sexual dysfunctions when patients and controls were compared. A positive correlation has been found between impulsivity, emotional dysregulation, oppositional symptoms severity and hypersexuality, risk-taking behavior, and sexual dysfunctions.

Finally, we published an original and outstanding review conducted by Ginapp et al., reporting about the lived experience of adults with ADHD. Thanks to their systematic literature review focused on the patients' subjective perceptions of the disorder, readers can realize the actual impact of ADHD core and accessory symptoms on daily life of people affected, even discovering some unexpected positive effects of impulsivity (e.g., spontaneous and funny attitude toward others) and attention deficit (e.g., creativity and motivating focus on details). The article yields a clear and comprehensive picture of the subjective experience of the person with ADHD also reporting about the huge effect of diagnosis and treatment.

## Author contributions

All authors contributed to manuscript revision, read, and approved the submitted version.

## Conflict of interest

The authors declare that the research was conducted in the absence of any commercial or financial relationships that could be construed as a potential conflict of interest.

## Publisher's note

All claims expressed in this article are solely those of the authors and do not necessarily represent those of their affiliated organizations, or those of the publisher, the editors and the reviewers. Any product that may be evaluated in this article, or claim that may be made by its manufacturer, is not guaranteed or endorsed by the publisher.
